# Evaluation of qPCR to Detect Shifts in Population Composition of the Rhizobial Symbiont *Mesorhizobium japonicum* during Serial in Planta Transfers

**DOI:** 10.3390/biology12020277

**Published:** 2023-02-09

**Authors:** Kenjiro W. Quides, Yoobeen Lee, Teresa Hur, Hagop S. Atamian

**Affiliations:** 1 Biological Sciences Program, Schmid College of Science and Technology, Chapman University, Orange, CA 92866, USA; 2 Department of Microbiology and Molecular Genetics, University of California, Davis, CA 95616, USA

**Keywords:** legume, rhizobium, passage, co-inoculation, symbiosis, qPCR, experimental evolution, nitrogen fixation, beneficial

## Abstract

**Simple Summary:**

Rhizobia species that fix atmospheric nitrogen in nodules that form on the root are very beneficial to plant growth and productivity. Highly efficient nitrogen-fixing rhizobia developed under laboratory conditions often do not function at the same level in natural soils with native microbial communities. The aim of this research is to study how the plant (*Lotus japonicus*) associates with three rhizobia from the same species (*Mesorhizobium japonicum)* when they are present together at the same time. These three rhizobia have different nitrogen-fixing abilities, making them highly beneficial (HB), moderately beneficial (MB), and non-beneficial (NM) to the plant. Using a quantitative PCR (qPCR)-based approach, we show that the plant selectively favors the HB rhizobia, such that at the end of the third generation, the plant was able to exclude almost all the MB and NB rhizobia from its nodules. To our surprise, when only the MB and NB rhizobia were present together, the plant could not favor the MB rhizobia and eliminate the NB rhizobia from its nodules. Studying more complex scenarios, with tens and hundreds of different rhizobia together, will help us better understand this interaction between rhizobia and plants and develop highly beneficial rhizobia to increase agricultural productivity.

**Abstract:**

Microbial symbionts range from mutualistic to commensal to antagonistic. While these roles are distinct in their outcome, they are also fluid in a changing environment. Here, we used the *Lotus japonicus*–*Mesorhizobium japonicum* symbiosis to investigate short-term and long-term shifts in population abundance using an effective, fast, and low-cost tracking methodology for *M. japonicum*. We use quantitative polymerase chain reaction (qPCR) to track previously generated signature-tagged *M. japonicum* mutants targeting the Tn5 transposon insertion and the flanking gene. We used a highly beneficial wild type and moderately beneficial and non-beneficial mutants of *M. japonicum* sp. nov. to demonstrate the specificity of these primers to estimate the relative abundance of each genotype within individual nodules and after serial transfers to new hosts. For the moderate and non-beneficial genotypes, qPCR allowed us to differentiate genotypes that are phenotypically indistinguishable and investigate host control with suboptimal symbionts. We consistently found the wild type increasing in the proportion of the population, but our data suggest a potential reproductive trade-off between the moderate and non-beneficial genotypes. The multi-generation framework we used, coupled with qPCR, can easily be scaled up to track dozens of *M. japonicum* mutants simultaneously. Moreover, these mutants can be used to explore *M. japonicum* genotype abundance in the presence of a complex soil community.

## 1. Introduction

Microbial symbionts and their hosts share a deep coevolutionary history that has resulted in multiple microbial mutualists. However, host organisms in these intimate interactions remain susceptible to antagonistic symbiont phenotypes. Antagonistic phenotypes could be the result of maladaptation [1] or opportunistic parasitic genotypes [2]. Nevertheless, hosts have evolved adaptive control mechanisms to mitigate the effects of antagonistic symbiont phenotypes [3,4]. Dynamic host control of diverse in vivo symbiont populations, commonly referred to as sanctions, is well described when ‘optimal’ symbionts are involved [5,6,7], but comparatively few studies have investigated the sanctions hypothesis with multiple suboptimal symbionts.

The legume–rhizobium symbiosis is a powerful system to investigate adaptive host control of symbiotic interactions and the underlying genetics of symbiont ecology and evolution processes [8]. For both host and symbiont, there are multiple genetic resources available, a well-established body of literature, and an abundance of biological materials. This includes multiple studies that have explored the interaction effects of host genotype, symbiont genotype, and environment [9]. However, the capacity to track the frequency of genotypic changes in longitudinal studies of symbiont populations has only recently become common practice with advances in sequencing technology [10,11,12]. Multi-generation experiments allow us to test the short-term impact of adaptive host control mechanisms and the potential for the long-term persistence of known rhizobial genotypes in artificial multi-genotype populations or complex microbial communities.

Rhizobia infection of legume roots, and persistence within, involves constant communication between soil-dwelling rhizobia and legumes [13]. Pre-infection communication consists of a suite of rhizobia nodulation genes [14]; exopolysaccharides play a large role during infection [15], and post-infection establishment involves an exchange of symbiotic metabolic resources [8]. This constant communication infers a series of molecular checkpoints throughout the interaction that a symbiont needs to pass to maintain access to legume resources, and failure to communicate can result in dramatic population size reduction through host-initiated premature nodule senescence [16,17]. Consequently, each checkpoint remains susceptible to rhizobia mutation that may allow evasion of host control and subsequently increase persistence and access to plant resources [8]. Nodules and plants are regularly coinfected by multiple strains of rhizobia [18], and adaptive host control mechanisms are constantly required to limit the persistence and proliferation of antagonistic rhizobial genotypes [8]. Here, we test multi-generation population shifts of multiple genotypes of *Mesorhizobium japonicum* in root nodules of *Lotus japonicus* by utilizing quantitative polymerase chain reaction (qPCR).

Culture-dependent methods have been demonstrated as reliable methods to track rhizobial symbionts, but these methodologies are limited to simple rhizobial populations or soil microbial communities. In addition, these methods are time-consuming and time-sensitive, requiring viable cells, while qPCR allows samples to be stored for future use to increase data collection during time-dependent plant dissections. Moreover, qPCR can distinguish between rhizobial genotypes that may be phenotypically indistinguishable via culturing. As molecular methods become increasingly affordable, qPCR offers a quick and efficient alternative to culturing techniques that can accommodate more complex experimental designs. However, there remain limitations to the utility of qPCR that need to be considered. Broadly, a standard qPCR protocol will not distinguish viable and non-viable cells, but viability workflows can mitigate these issues [19]. For rhizobia specifically, many species undergo terminal genome duplication (i.e., mostly those forming indeterminate nodules [20]), which can interfere with qPCR estimates and indicators of serial infection potential. In this study, we use previously generated near-isogenic Tn5 signature-tagged *M. japonicum* sp. nov. mutants [21] with known insertion points that form determinate nodules on *Lotus japonicus* and remain viable after natural nodule senescence [17,22,23]. In this work, we use qPCR to track the rhizobial symbionts in vitro, in planta, and over time to study the relative fitness effects of single-gene knockouts.

## 2. Materials and Methods

### 2.1. Biological Materials

*Lotus japonicus* MG-20 seeds were acquired from LegumeBase (National BioResource Project; University of Miyazaki, Miyazaki, Japan) and grown in a growth chamber (CARON Products; Marietta, OH, USA; PAR: 300umolm-2s-1; humidity: 65%; temp: 22 °C; 16 h light:8 h dark).

We used three near-isogenic *Mesorhizobium japonicum* sp. nov. genotypes that provide varying levels of growth benefits to *L. japonicus* [23]. The beneficial wild type genotype has a unique DsRed marker integrated into the genome, resulting in red colonies under natural light. The moderately beneficial genotype (STM30; ORF mll0343, GSI; strain ID 10T05g06) provides ~50% of the shoot growth benefit compared to plants clonally inoculated with the wild type, and the non-beneficial genotype (STM6; mlr5906, nifD; 17T02d02) provides a benefit equivalent to an uninoculated plant, but the in vitro growth rates and colony morphology (excluding color) of these two genotypes are indistinguishable from the wild type [23]. Both mutants were previously generated using signature-tagged mutagenesis and have a Tn5 insertion at known positions [21]. All *M. japonicum* were grown on solid modified arabinose gluconate (1.8% *w*/*v*, 29 °C) [22]. 

### 2.2. Standard Curve

Standard curves were generated for each of the three *M. japonicum* genotypes to correlate colony-forming units to cell density estimates via qPCR (Code S1). Seven replicates of clonal cell suspensions (~10^9^–10^10^ cells mL^−1^) were created for each genotype. Stock cell suspensions were serially diluted and spread-plated (10^−8^) to estimate colony-forming units (CFU). The stock solution, 10^−2^ dilution, and 10^−4^ dilution were saved for DNA extractions and qPCR cell density estimates. CFU counts were scaled to their appropriate qPCR dilution when creating standard curves.

### 2.3. In Vitro Experiment

Clonal plates of *M. japonicum* were grown to lawns (3–5 days; 29 °C) before washing and resuspending in sterile double distilled water (ddH_2_O) to a density of ~10^10^ cells mL^−1^ estimated by OD_600_. For each wild type and Tn5 mutant combination, we created eight cell suspensions with an estimated 10–90% of wild type to correlate CFU proportion estimates with qPCR proportion estimates. Co-suspensions were diluted as above, with the 10^−2^ dilution used for qPCR ratio estimates and the 10^−8^ dilution used for CFU ratio estimates. CFU ratios were determined by counting red (DsRed wild type) vs. cream (Tn5 mutants) colonies on up to three plates. Two technical replicates of each sample were estimated by qPCR.

### 2.4. In Planta Experiment

Individual nodules were randomly sampled from the passaging experiments (see below) generated by co-inoculating seedlings with the wild type and either mutant. We compared CFU and qPCR estimates of wild-type proportion for 93 nodules of each co-inoculation (186 nodules total). The nodules were a combination of white (ineffective) and pink (effective). White nodules were all similar in size (<1 mm). Pink nodules were both small (<1 mm) and large (>1 mm). Nodules were independently surface sterilized in bleach for 30–60 s, rinsed three times in sterile ddH_2_O, crushed with a sterile pestle, and resuspended in 1000 µL of sterile ddH_2_O. The nodule suspension was spread-plated across three plates at two different dilutions to capture the range of cell densities (10^4^ and 10^5^). Colonies were counted, and CFU proportions were determined by color. DNA was extracted from the crushed nodule suspension, and genotype proportion was estimated using qPCR. Although these 186 nodules were sourced from the passaging experiments, the rhizobia within were excluded from further passaging.

### 2.5. Passaging Experiment

- Germination Pouches:

*Lotus japonicus* MG-20 seedlings were grown in CYG germination pouches (Mega International; Newport, MN, USA) following a modified published protocol [23,24]. Briefly, surface sterilized seeds were nick scarified with a scalpel and germinated and maintained in CYG germination pouches filled with N-free Jensens fertilizer [25]. Bundles of five pouches were wrapped in aluminum foil to limit light to the roots and placed in clear plastic boxes with ventilation holes to maintain humidity and limit fungal contamination (Figure S1). Each pouch received five seeds, with the first pouch left empty due to accelerated desiccation (20 seeds per bundle). Plants were maintained in a growth chamber with the same settings as above (Section 2.1).

We tested the competitive ability of the more beneficial genotypes [23] by tracking six different combinations of *M. japonicum* types and ratios through three passages of in planta growth. *L. japonicus* MG-20 seedlings were inoculated 10 days after scarification when one true leaf had emerged. The plants were inoculated with six different co-inoculation ratios estimated by OD_600_. The ratios included wild type:STM30 (1:1), wild type:STM30 (1:9), wild type:STM6 (1:1), wild type:STM6 (1:9), STM30:STM6 (1:1), and STM30:STM6 (1:9). A total of 5 × 10^7^ cells in 50 µL of cell suspension from each combination was dripped directly on the root system of each plant individually. Plants were grown for four weeks, and only distinctly spherical nodules with separation from the root were harvested for data collection and the next inoculation.

The second set of plants was inoculated with rhizobia extracted from the nodules of the previous set of plants. Nodules from a given co-inoculation ratio were counted and removed. Nodules were surface sterilized by vortexing in bleach (2–3 min) and rinsed seven times with sterilized ddH_2_O (1 min). Sterile nodules were crushed with a sterile pestle and resuspended in 3 mL of sterile ddH_2_O. A subset of nodule suspension was set aside for populations initiated with the wild type to compare cell density estimates via CFU and qPCR, as above. The remaining crushed nodule suspension was used to inoculate the next set of plants. The cell density of the crushed nodule suspension was not standardized to facilitate the rapid transfer of rhizobia (i.e., no in vitro growth step or cell density measurement prior to inoculation). Plants were grown one more cycle for a total of three rounds of rhizobia exposure to in vivo growth (total of 3 passages). Each co-inoculation ratio lineage was exposed to plants from two bundles of plants at each passage (up to 40 plants). All plants within two bundles inoculated with the same co-inoculum represented an individual passage line with all nodules from those bundles passaged to two new pre-assigned bundles of plants.

b. Magenta Boxes:

Seedlings for new passage lines were grown in double-stacked magenta boxes (PlantMedia; Dublin, OH, USA; Figure S1) [26] to simulate a natural growth setting and increase the number of lineages within the growth chamber. The bottom box was filled with 500 mL of 50% strength N-free Jensen’s fertilizer, and the top box was filled with calcined clay within three cm from the top (Turface Pro League, Turface Athletics, Buffalo Grove, IL, USA). A 13 mm hole was cut through the base of the top box to connect fertilizer and clay with a 20 cm long, 6 mm diameter cotton wick held in place by a 3D printed nylon stopper (Taulman Nylon 680, Taulman3D, St Peters, MO, USA). All boxes were assembled prior to autoclave sterilizing. Each box received five bleach-sterilized and scarified seeds to maximize the number of nodules formed while limiting seedling root competition in magenta boxes. Plants were maintained in a growth chamber with the same settings as above (Section 2.1).

Seedlings were inoculated 10 days after planting when one true leaf had emerged. We tracked a total of 36 independent population lineages as follows: wild type:STM30 (1:9 ratio; 3 lineages), wild type:STM6 (1:9; 3 lineages), STM30:STM6 (1:1; 10 lineages), STM30:STM6 (1:9; 10 lineages), and wild type:STM30:STM6 (1:1:1; 10 lineages). We put the wild type at an initial competitive disadvantage in co-inoculation to emphasize competitive ability, and we increased the number of independent population lineages in passages containing both STM30 and STM6 because these combinations have not previously been tested for shifts in genotype proportion. Rhizobial populations were passaged as above with three exceptions: (1) plants were inoculated with 500 µL of cell suspension (initial inoculation at a concentration of 10^9^ cells mL^−1^; 5 × 10^8^ cells total) dripped around the base of each seedling, (2) crushed nodules were resuspended in 8 mL of sterile ddH_2_O, and (3) each lineage was exposed to a maximum of 15 plants at each passage (i.e., three boxes of plants). Our OD_600_ measurements for STM30:STM6 and wild type:STM30:STM6 were less accurate than expected, which resulted in a wide variation of initial inoculation ratios for these lineages. For STM30:STM6 combinations, all 20 lineages (10 × 1:1 and 10 × 1:9) were pooled for analyses.

### 2.6. DNA Extraction

DNA was extracted using a cetyltrimethylammonium bromide method (CTAB) [27]. A total of 100 µL of the sample (cell suspension or crushed root tissue) was mixed with 400 µL of CTAB buffer (2% CTAB *w*/*v*, 1.4 M NaCl, 100 mM Tris, 20 mM EDTA) and incubated for 30 min at 65 °C. Samples were cooled to room temperature (RT), and 600 µL of chloroform was added. Samples were vortexed, incubated at RT for 2 min, and centrifuged at 12,000× *g* for 15 min at RT. Then, 200 µL of supernatant was transferred to a new tube with 400 µL of 100% ethanol (EtOH). Samples were incubated at −20 °C for 30 min before centrifuging at 14,000× *g* for 15 min at 4 °C to pellet DNA. Pelleted DNA was washed with 600 µL of 70% EtOH at 10,000× *g* for 5 min at RT. Washed pellets were allowed to air dry before resuspending in 50 µL of nuclease-free water.

### 2.7. qPCR Assay

We designed specific primers for the two Tn5 mutants in this study, which included one primer annealing to the Tn5 insertion [28], with the second primer designed to anneal to the flanking genome region (Table S1). For the wild-type phenotype *M. japonicum*, we utilized a strain containing DsRed integrated into the genome (M. Hayashi’s personal communication; [29]), which allowed us to use DsRed primers to identify the wild type [30]. These primers were used throughout to estimate the proportions of each genotype in vitro and in planta via qPCR (Table S1).

qPCR was performed in a total volume of 25 µL. Each reaction contained 1 µL of template DNA, 1× SYBR Green qPCR master mix (Bio-Rad; Hercules, CA, USA), and primers at a final concentration of 0.4 µM. Reactions were run on a BioRad CFX96 Real-Time System C1000 Touch Thermocycler with the following settings: initial warm-up at 95 °C for 3 min followed by 40 cycles of denaturation at 95 °C for 15 s, annealing at 58 °C for 20 s, and extension at 72 °C for 30 s. A melt curve was generated from 65 °C to 95 °C with an increase of 0.5 °C every second.

### 2.8. Data Analysis

All analyses and visualization were performed in R (v4.1.2; Code S1). Colony-forming unit (CFU) population estimates were scaled to cells per nodule, which was equivalent to cells mL^−1^ (i.e., each nodule was resuspended in 1 mL of sterile H_2_0). CFU proportions of wild type to mutant were calculated according to colony color for all spread-plated replicates. Quantitative polymerase chain reaction (qPCR) population estimates were based on a standard curve created for each genotype (Code S1). qPCR samples that did not reach a cycle threshold (CT) were assigned a value of 40 (i.e., the maximum number of cycles). CT values less than negative controls for each primer pair in a 96-well plate were used to calculate cell estimates. All other CT values were excluded from further analyses. Mean cell estimates were calculated for a given sample, and proportions were estimated based on these mean values. CFU and qPCR methodology were compared using linear regression for fit and Bland–Altman analysis to measure differences.

## 3. Results

### 3.1. qPCR and CFU Estimates of Genotype Proportions Strongly Correlate In Vitro and in Planta

#### 3.1.1. In Vitro

The qPCR estimates were strongly correlated with our CFU proportion estimates (wild type:STM30—R2 = 0.98; wild type:STM6—R2 = 0.97; Figure S2). The Bland–Altman comparison of differences indicated qPCR proportion estimates were 0.068 less than CFU estimates and 0.111 greater than CFU estimates for wild-type co-suspensions with STM30 and STM6, respectively (Figure S3).

#### 3.1.2. In Planta

**Figure 1:**
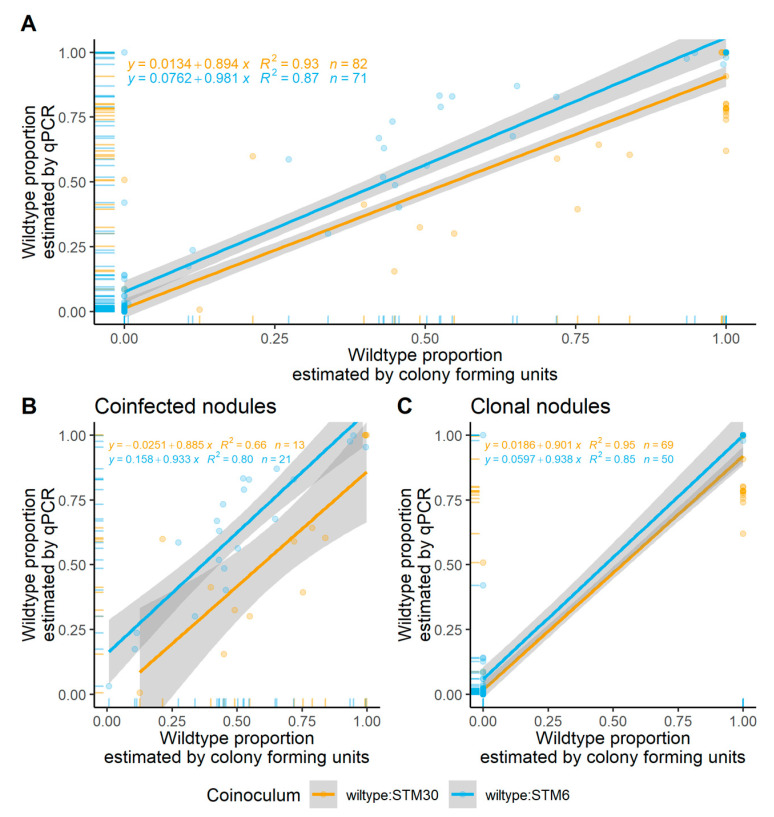
Colony forming units vs. quantitative polymerase chain reaction in planta. The proportion of the wild-type strain was estimated in planta. Wild type was distinguished from the Tn5 mutant using DsRed. DsRed expression is visible under natural light when counting CFUs and provides a specific target for qPCR. Tn5 mutants were identified with qPCR using specific primers targeting the Tn5 transposon and flanking gene region. Linear models are provided for all nodules (**A**), coinfected nodules detected by CFU (**B**), and clonally infected nodules estimated by CFU (**C**) with 95% confidence intervals included (grey) for each set of samples. Each data point represents a proportion estimated for a single nodule. The size of the data point indicates the estimated viable population size by CFU. Marginal plots indicate the number of data points along each axis. Orange = wild type:STM30 samples, and blue = wild type:STM6 samples.

Due to the variable nature of nodulation, we were unable to collect data across a consistent range of proportions, with most of our data points occurring at the extremes (Figure 1 and Figure S4). Overall, we found the proportions to be comparable to CFU estimates (wild type:STM30—R2 = 0.93; wild type:STM6—R2 = 0.87). Bland–Altman comparisons of qPCR estimates were 0.037 less than CFU estimates and 0.071 greater than CFU estimates for nodules formed by the wild type co-inoculated with STM30 and STM6, respectively (Figure S3).

Of the 93 random nodules sampled from the wild type:STM30 and wild type:STM6 combination lineages, we successfully cultured and conducted qPCR for 82 and 71 nodules, respectively (Figure 1A; Table S1). A total of 16% of wild type:STM30 (13/82) generated nodules were coinfected, and 30% of wild type:STM6 (21/71) nodules were coinfected when estimated by CFU with support from qPCR to accurately estimate coinfection (Figure 1B).

When estimated by CFU, 40 total nodules were clonally infected with the wild-type genotype and 79 were clonally infected with either mutant from their respective populations (Figure 1C). Clonal nodules generated by a wild type:STM30 population were 45% wild type (31/69) and 55% STM30 (38/69). In nodules generated from a wild type:STM30 population, qPCR estimated the wild type dominated the nodules (>99%) in 19 of 31 nodules, with no values lower than 61%. Additionally, from STM30 nodules, qPCR estimated the mutant represented >99% of the nodule population for 31 of 38 nodules, with no values lower than 71%. Clonal nodules generated by a wild type:STM6 population were 18% wild type (9/50) and 82% STM6 (41/50). In nodules generated from wild type:STM6, only one sample was estimated at under 99% wild type (98%). Lastly, qPCR estimated 16 of 41 nodules had populations of >99% STM6, with all but three nodules estimated to be >90% (10%, 42%, and 100%). In total, CFU estimated the wild type was present in 44 of 82 nodules, STM30 was present in 51 of 82 nodules from wild type:STM30-generated nodules, the wild type was present in 30 of 71 nodules, and STM6 was present in 59/71 nodules from wild type:STM6-generated nodules.

### 3.2. Application for Tracking Shifts of Rhizobial Populations upon Serial Transfers in Planta

#### 3.2.1. Germination Pouches

**Figure 2:**
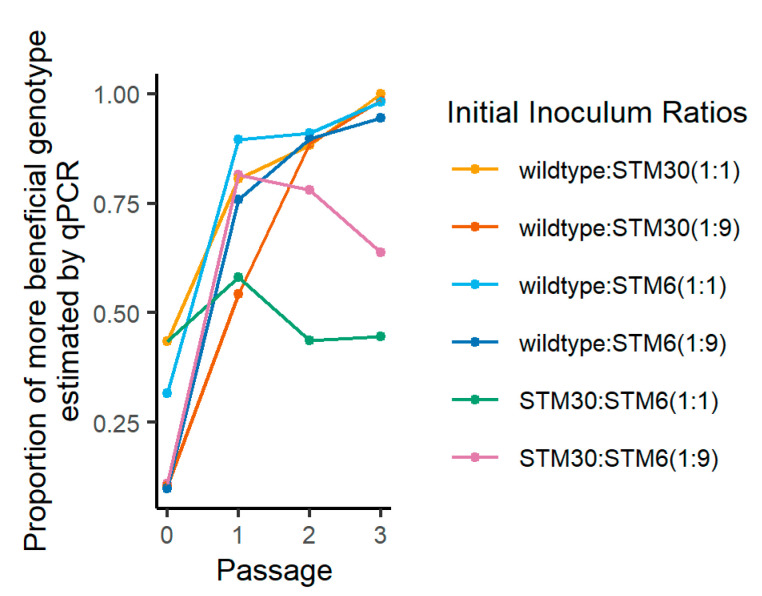
Change in proportion of more beneficial genotype over serial exposure to in planta growth in germination pouches. The proportion of the more beneficial genotype was estimated using qPCR at four different time points: the initial inoculation and after passage 1, 2, and 3 of in planta growth. Each combination of rhizobia genotypes had a target initial inoculation ratio of 1:1 and 1:9 (more beneficial:less beneficial). The order of benefit provided by rhizobial symbionts is wild type > STM30 > STM6.

We tracked simple populations of *M. japonicum* through multiple exposures to in planta growth (Figure 2 and Figure S5), and these results were comparable when estimated via qPCR and CFU (R2 > 0.81; Figure S6). We found the wild type increased in the in planta proportion of the population over time (Figure 2) [23]. When the two mutant genotypes, STM30 and STM6, were co-inoculated, neither mutant was fixed in either lineage (Figure 2). Our passaging results were consistent with a previous study using these rhizobial genotypes, and we found no indication that DNA from non-viable cells led to inaccurate proportion estimates (i.e., wild type consistently increased in relative abundance; [23]).

#### 3.2.2. Magenta Boxes

**Figure 3:**
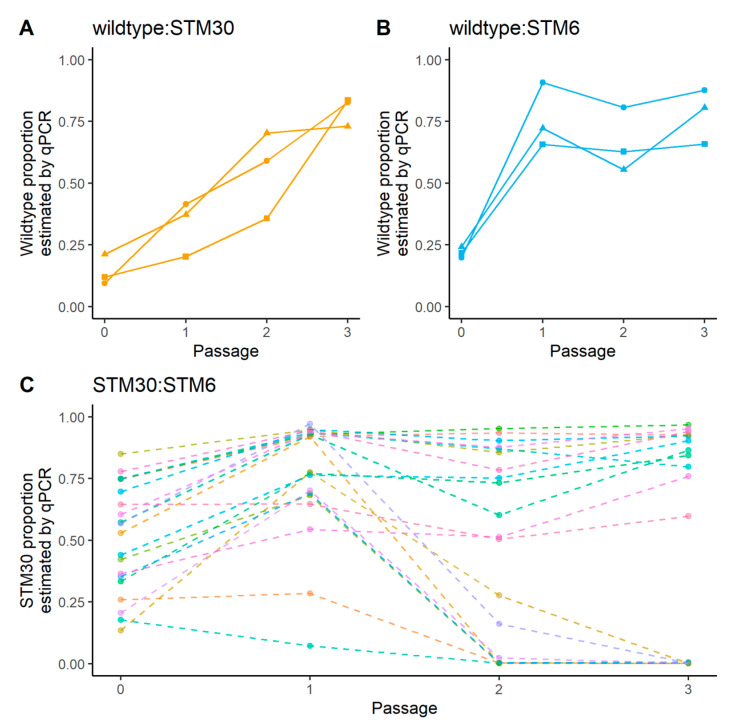
Change in proportion of more beneficial genotype over serial exposure to in planta growth in magenta boxes. The wild type was co-inoculated in three independent lineages with STM30 (**A**) and STM6 (**B**). The two mutants were co-inoculated together in 20 independent lineages (**C**). The proportion of the more beneficial genotype was estimated using qPCR at four different time points: the initial inoculation and after passages 1, 2, and 3 of in planta growth. The different colors in (**C**) represent the 20 lineage replicates of STM30 and STM6. The order of benefit provided by rhizobial symbionts is wild type > STM30 > STM6.

**Figure 4:**
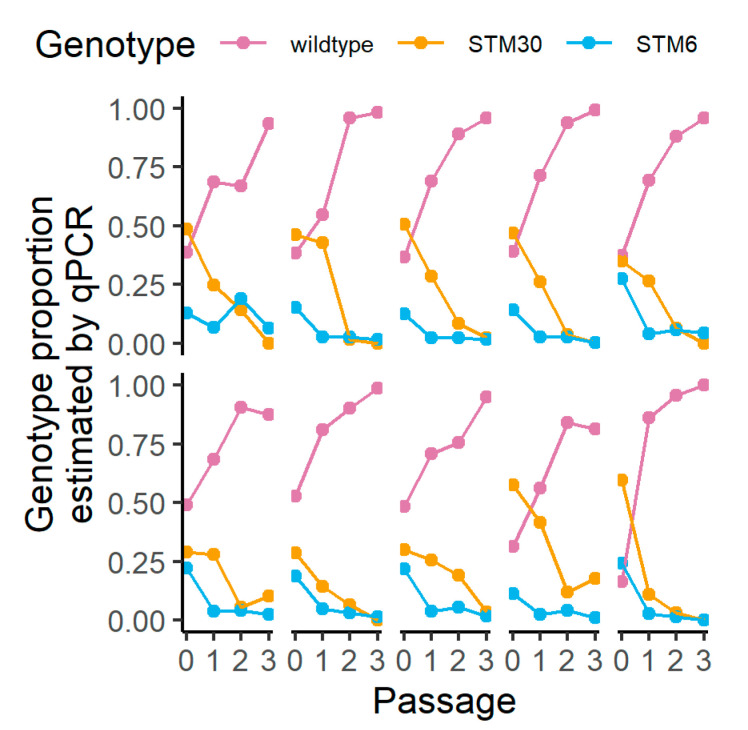
Change in proportion of three genotypes over serial exposure to in planta growth in magenta boxes. Ten independent lineages with initial inoculations containing wild type, STM30, and STM6 genotypes were passaged and global changes in the proportion of each genotype were modeled over passage time. The proportion of the more beneficial genotype was estimated using qPCR at four different time points: the initial inoculation and after passages 1, 2, and 3 of in planta growth. The order of benefit provided by rhizobial symbionts is wild type > STM30 > STM6. Pink = wild type, orange = STM30, and blue = STM6.

We altered the growth method to more closely mimic natural root growth in 36 new population lineages to investigate population dynamics when both mutants were present. First, we confirmed the wild type increased in proportion, despite an initial population size disadvantage, over time using this new growth methodology (Figure 3A,B and Figure S7). With 20 lineage replicates of STM30 and STM6 co-inoculated onto initial hosts, we did not find support for a consistent increase in one genotype over the other (Figure 3C and Figure S8). We also tracked 10 lineages of all three genotypes and found the wild type increased in the proportion of the population over time, while both mutants consistently decreased in the proportion of the population over time (Figure 4 and Figure S9).

## 4. Discussion

We demonstrated qPCR as an alternative method to track the genotypes of the near-isogenic *M. japonicum* used in this study. We found a strong correlation of genotype proportion estimates between CFU and qPCR (R2 > 0.81), and the wild type consistently increased in our passaging experiments, similar to previous work with these genotypes [23]. Additionally, the use of qPCR allowed us to track a dual-genotype population that cannot be distinguished on plates (i.e., STM30 and STM6). Lastly, qPCR allowed us to increase the scale of this methodology to track individual genotypes over multiple exposures to in planta growth phases. In total, we tracked 42 lineages of rhizobia, which could have approached 100 independent lineages in our experimental timeframe without simultaneous culturing. This methodology provides the framework to track multiple genotypes of *M. japonicum* sp. nov. [21] in increasingly complex populations (i.e., with other mutants) or communities (i.e., with a soil microbiome) as we disentangle fitness trade-offs (host dependent or independent) between symbiont genotypes in different environmental contexts.

Following individual rhizobia species after serial exposures to new legume hosts has become increasingly practical in recent years [31,32,33]. Select and resequence has been demonstrated as a powerful technique to study rhizobial evolution in various environments [34] and over multiple generations [35]. Similarly, qPCR has been used to track diverged rhizobial species over multiple generations [35]. Both of these studies utilized the *Medicago truncatula*-*Ensifer meliloti* system [34,35], and our approach is an ideal complement using *L. japonicus,* which forms determinate nodules that house quantifiable viable rhizobia that can infect a new set of legume roots [8]. Additionally, the signature-tagged *M. japonicum* we used is near-isogenic, allowing for highly controlled ecological and evolutionary experiments with single-gene knockout mutants [21].

Unlike Batstone et al. (2020) [35], which used diverged rhizobial species, in this study, we utilized two near-isogenic mutants that vary in the symbiotic benefit provided to the host. Previous work has shown both STM30 and STM6 are outcompeted by the wild-type rhizobia, but the competition between the two mutants was not investigated [23]. In agreement with the previous work, the wild type consistently overtook populations when present in our passaging experiments. This occurred regardless of starting population proportion, growth method, and the number of mutants present. In the pouch experiments, starting ratios (1:1 or 1:9, wild type:mutant) had no impact on the proportion of the population after three passages. In magenta boxes, the wild type never started at greater than 25% of the population but steadily increased with STM30 and sharply increased with STM6. The relative abundance of the wild type after three passages was not as high as in pouches, suggesting the potential for the greater persistence of suboptimal symbionts in a soil-like setting. Similarly, when tri-inoculated in magenta boxes, the wild type performed similarly to co-inoculation, but the mutants followed a slight trend based on the benefit provided. STM6 experienced a rapid population decline, while the decline with STM30 was more gradual.

The sanctions hypothesis predicts that STM30 should outcompete STM6 in planta [5,23], but our passaging experiments did not find a consistent pattern of STM30 fixing in the population, and STM6 was the dominant genotype in multiple lineages at the end of three passages (Figure 3C) [23]. Although we did not culture individual nodules from STM30:STM6 inoculated plants, individually cultured nodules with either mutant or the wild type suggest a reproductive trade-off. Despite sanctions at the plant level, as shown through passaging, both mutants persisted within nodules. STM6 was present in ~87% of nodules sampled, and STM30 was present in ~62% of nodules sampled. While STM6 nodules likely experienced cell-autonomous sanctions due to a lack of nitrogen fixation, the corresponding accumulation of host carbon as polyhydroxybutyrate (PHB) [17], a vital energy storage molecule for rhizobia [36], would have provided a reproductive boon. Conversely, STM30 provides suboptimal benefit, but this benefit could still result in nitrogen fixation and in planta proliferation [23,37]. Thus, the availability of carbon for rhizobial reproduction in STM30 and STM6 might dictate which genotype attains greater relative abundance in a dual-genotype population, but factors that influence carbon availability in this scenario were not explored. Our data suggest that 50% benefit provided (STM30; relative to wild type) does not provide carbon resources sufficient to regularly outcompete a symbiont that redirects host carbon to store PHB (STM6). This reproductive trade-off between moderate nitrogen fixation, thus moderate carbon reciprocated, and hoarding host-derived carbon is just one example of the intricate nature of this symbiosis to be further investigated.

The use of qPCR to assess shifts in rhizobial abundance has limitations compared to traditional culturing techniques, but it also has advantages. The most apparent limitation is the inability to distinguish the viability of cells. From a technical perspective, there are workflows to mitigate these issues [19], which would be useful when individually assessing nodules. Depending on the goal of the experiment, the vastly increased number of nodules that can be processed by qPCR may be worth the additional noise that we experienced. For example, 3 out of 41 clonally infected STM6 nodules have markedly different CFU and qPCR results. This may have been a result of the amplification of DNA from non-viable cells or the inactivation of DsRed. Additionally, there may be differences in cells released during the crushing of nodules and lysis of cells for DNA extraction. However, the multi-generation experimental approach used here avoids issues of cell viability by tracking over time, therefore linking viability to infectability, and mitigates bottlenecking by using a large pool of nodules. Lastly, the most powerful aspect of qPCR in this study is that it allows us to distinguish genotypes that are indistinguishable by culturing techniques, such as those from the >6500 genotype library used here [21]. Future work offers an opportunity to explore the effect of these single-gene knockouts on bacterial populations in a dynamic soil environment as we continue to grapple with microbiome applications [33].

## 5. Conclusions

The *M. japonicum* mutants we used here provide an accessible opportunity to investigate genes that affect rhizobia in symbiosis, free-living, or both. The Tn5 primers are easily adaptable, and complementary primers in flanking regions can be readily designed. These specific tags allow for reliable tracking mechanisms of genotypes that could be optimized for various stages of rhizobia life. In populations with near-isogenic *M. japonicum*, we could design experiments that identify genes that influence selection at various life stages. Additionally, we could attempt to track gene-knockout mutants in competitive soil microbiomes. The effective, fast, and low-cost tracking of rhizobial genotypes will allow us to ask guided questions that further our understanding of the mutualism-antagonism continuum in the legume-rhizobium symbiosis and identify genotypic synergism in the soil microbiome.

## Data Availability

The data supporting reported results can be found in the Supplementary Materials file SuppData.xlsx. The code used for the analysis of all the figures can be found in the file SuppCode.R.

## Supplementary Materials

The following supporting information can be downloaded at: https://www.mdpi.com/article/10.3390/biology12020277/s1, Figure S1: *Lotus japonicus* growth setup; Figure S2: Colony-forming units vs. quantitative polymerase chain reaction in vitro; Figure S3: Bland–Altman plots to compare population proportion estimates by colony-forming units and quantitative polymerase chain reaction; Figure S4: Colony-forming units vs. quantitative polymerase chain reaction in planta; Figure S5: wild type and mutant qPCR cell estimates while passaging in growth pouches; Figure S6: Passaging in germination pouches; Table S1: Primers used in this study, all the data generated in this study; Figure S7: Wild type and mutant qPCR cell estimates while passaging in magenta boxes; Figure S8: Mutant qPCR cell estimates while passaging in magenta boxes; Figure S9: Wild type and both mutant qPCR cell estimates while passaging in magenta boxes. Code S1: The R scripts used for data analysis and generating figures.
