# Supplementary material for: Evaluation of qPCR to Detect Shifts in Population Composition of the Rhizobial Symbiont Mesorhizobium japonicum during Serial in Planta Transfers
Source: Biology (Basel). 2023 Feb 9;12(2):277. doi: 10.3390/biology12020277 (PMC9953586; doi:10.3390/biology12020277)
Supplement: Supplementary file 1 [file biology-12-00277-s001.zip › biology-2128511- Supplementary Figures.pdf]

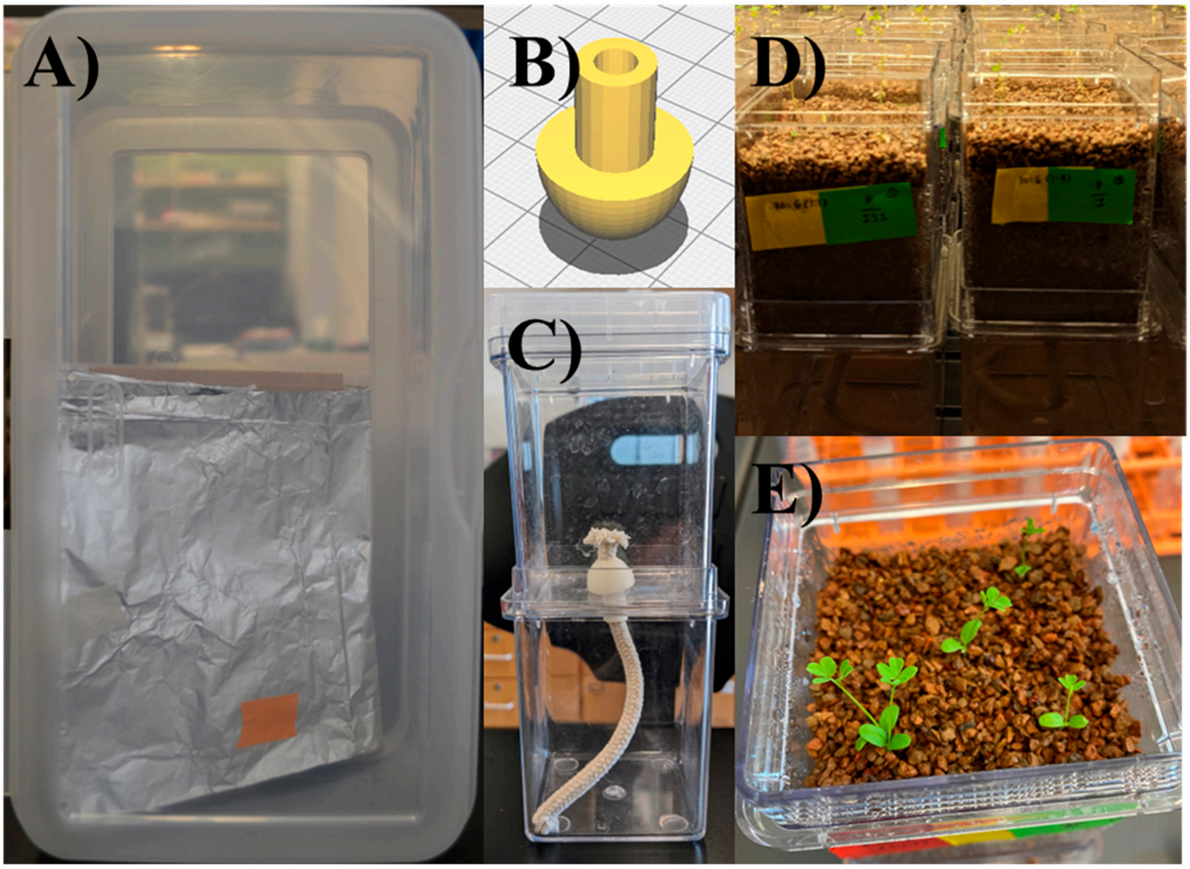

Figure S1. ***Lotus japonicus*** **growth setup.** Bundles of germination pouches (A) and stacked magenta boxes were used for plant growth (C). Fertilizer was added directly to pouches to wick nutrients to plant roots or to the bottom box which was transferred to clay in the top box using a cotton wick held in place with a 3D printed nylon plug (B). Seedlings were germinated and maintained in a growth chamber (D) and inoculated after 10 days when one true leaf emerged (E).

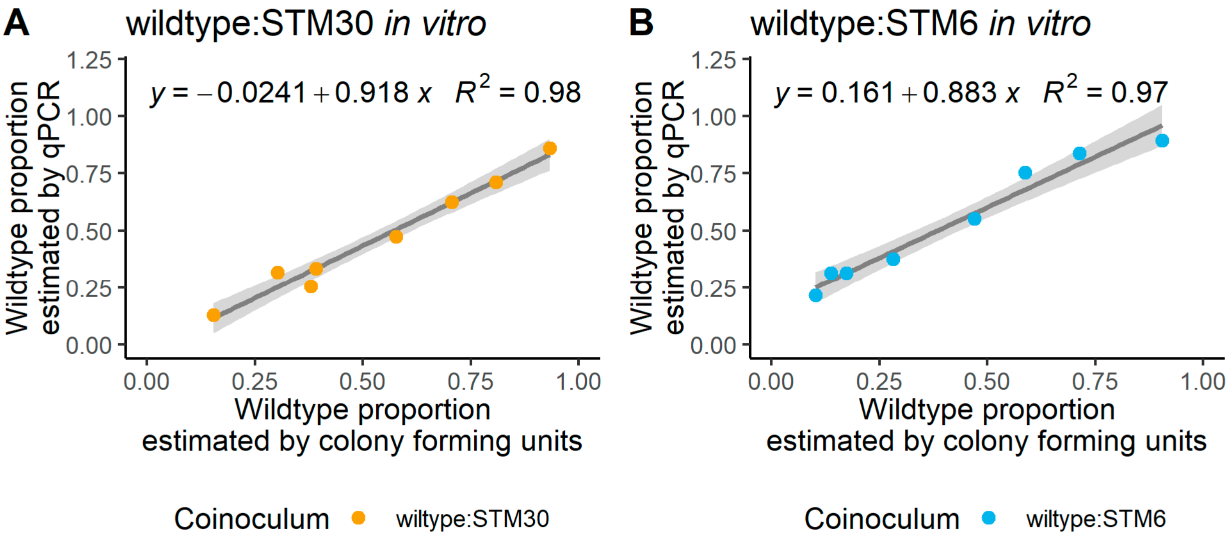

Figure S2. Colony forming units vs. quantitative polymerase chain reaction *in vitro*. Proportion of the wildtype was estimated *in vitro*. Wildtype was distinguished from the Tn5 mutant using DsRed. DsRed expression is visible under natural light when counting CFUs and provides a specific target for qPCR. Tn5 mutants were identified with qPCR using specific primers targeting the Tn5 transposon and flanking gene region in STM30 (A) and STM6 (B). Each data point represents an artificially mixed cell suspension. Orange = wildtype:STM30 samples and blue = wildtype:STM6 samples.

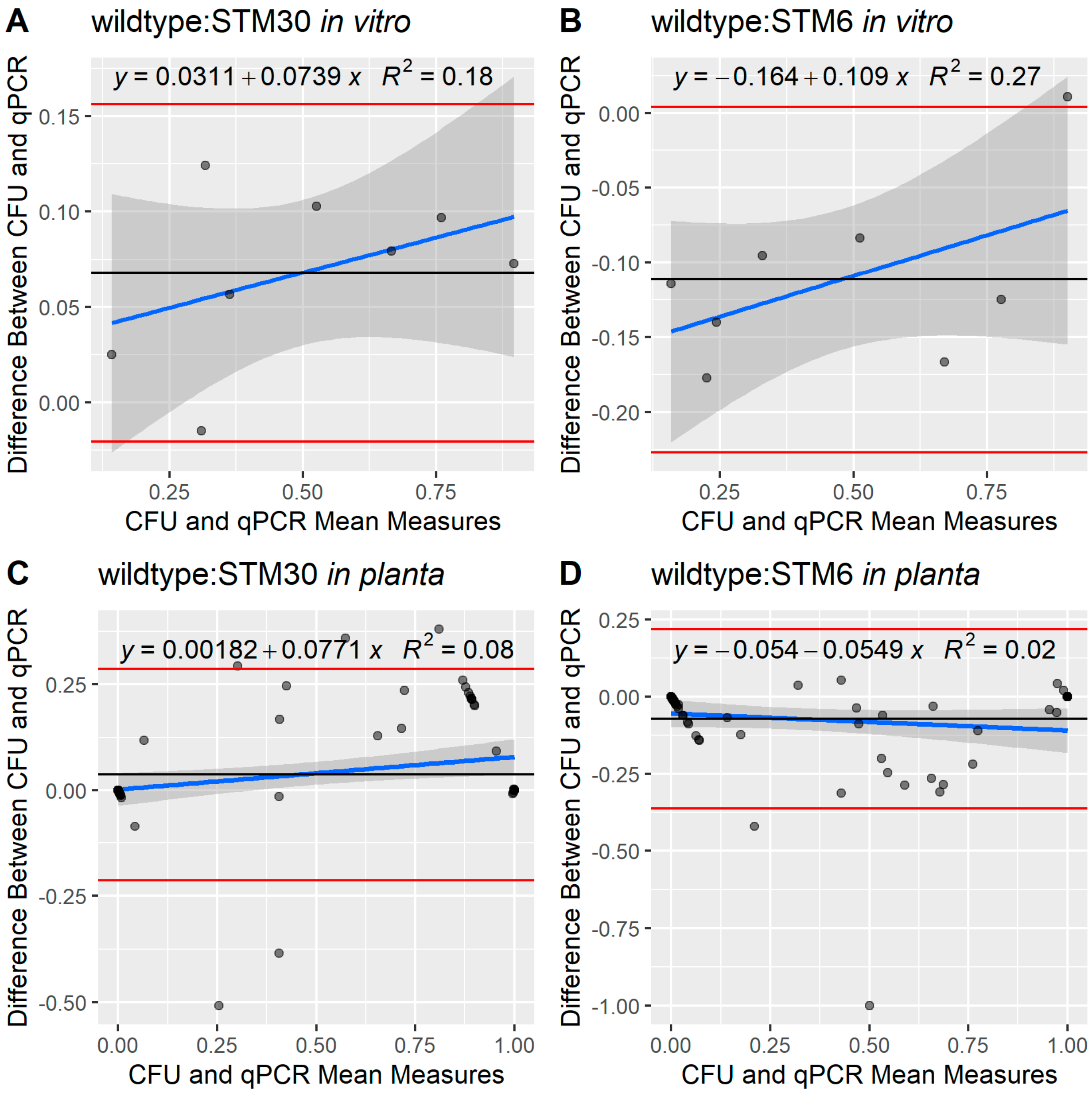

Figure S3. Bland-Altman plots to compare population proportion estimates by colony forming units and quantitative polymerase chain reaction. Plots were created for *in vitro* (A, B) and *in planta* (C, D) comparisons for samples containing wildtype and STM30 (A, C) or STM6 (B, D). Horizontal black lines represent the mean difference between methods (CFU – qPCR) and red lines are 1.96 standard deviations away from the mean. Linear models indicate potential for biases in methodology differences for mean measures.

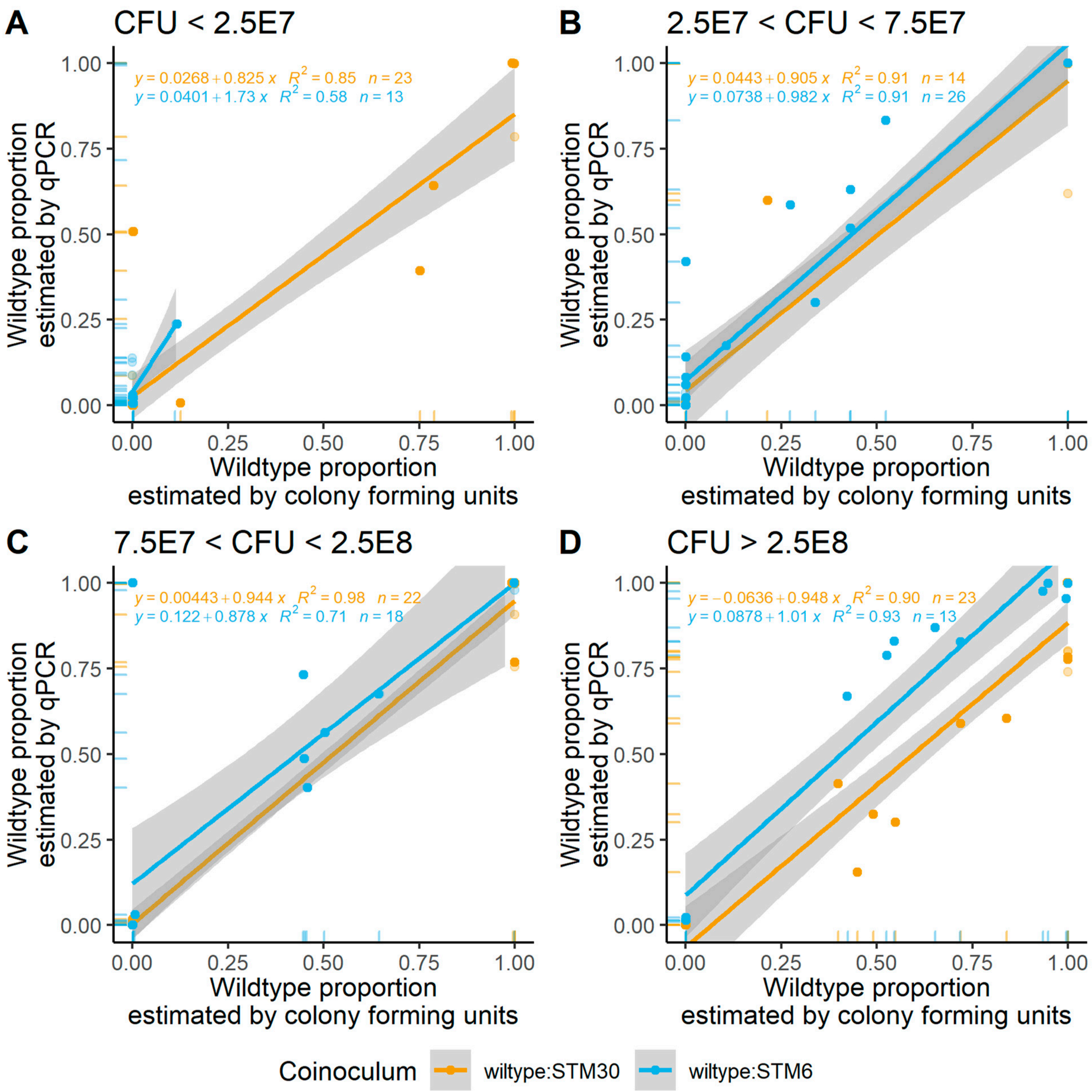

Figure S4. Colony forming units vs. quantitative polymerase chain reaction *in planta*. Linear models were independently generated for subsets based on cell density estimated by CFU. Nodules with less than 2.5*10^7 viable cells (A), 2.5*10^7 – 7.5*10^7 viable cells (B), 7.5*10^7 – 2.5*10^8 viable cells (C), and greater than 2.5*10^8 viable cells. Each data point represents a proportion estimated for a single nodule. Marginal rug plots indicate number of data points along each axis. Orange = wildtype:STM30 samples and blue = wildtype:STM6 samples.

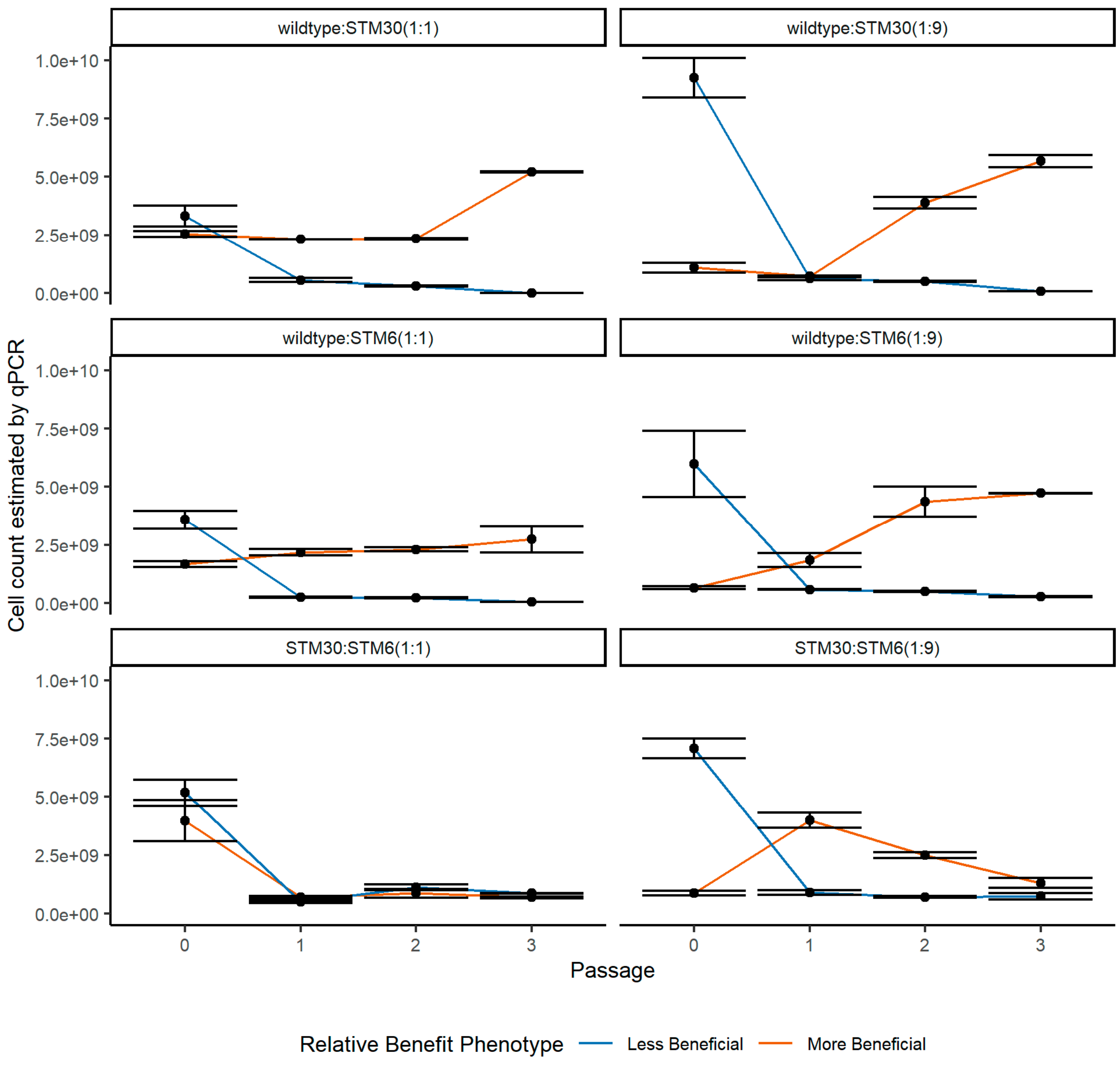

Figure S5. Estimated population sizes of rhizobia using qPCR over serial exposure to *in planta* growth in germination pouches. The population size of each genotype was estimate using qPCR at four different time points: the initial inoculation and after passage 1, 2, and 3 of *in planta* growth. Seedlings were inoculated with a high concentration of rhizobia in ratios of 1:1 and 1:9 (more beneficial:less beneficial). The order of benefit provided by rhizobial symbionts in wildtype > STM30 > STM6. Error bars indicate standard error of qPCR technical replicate estimates.

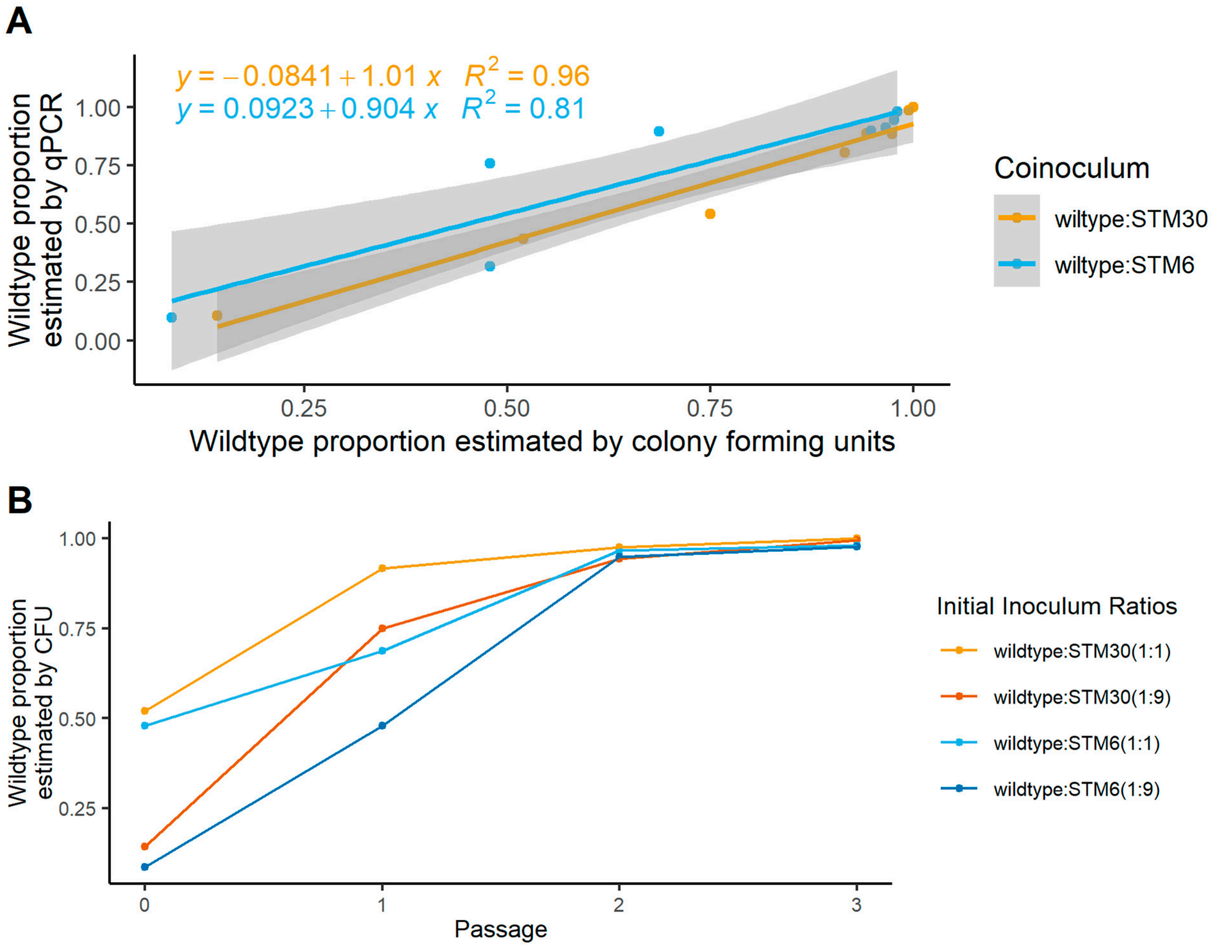

Figure S6. Passaging in germination pouches. Estimates of the wildtype proportion measured by colony forming units and quantitative polymerase chain reaction were correlated for each sample collected from passages 0, 1, 2, and 3 (A). The proportion of the wildtype estimated by colony forming units consistently increased over passage time (B). The wildtype was coinoculated with STM30 (warm colors) and STM6 (cool colors).

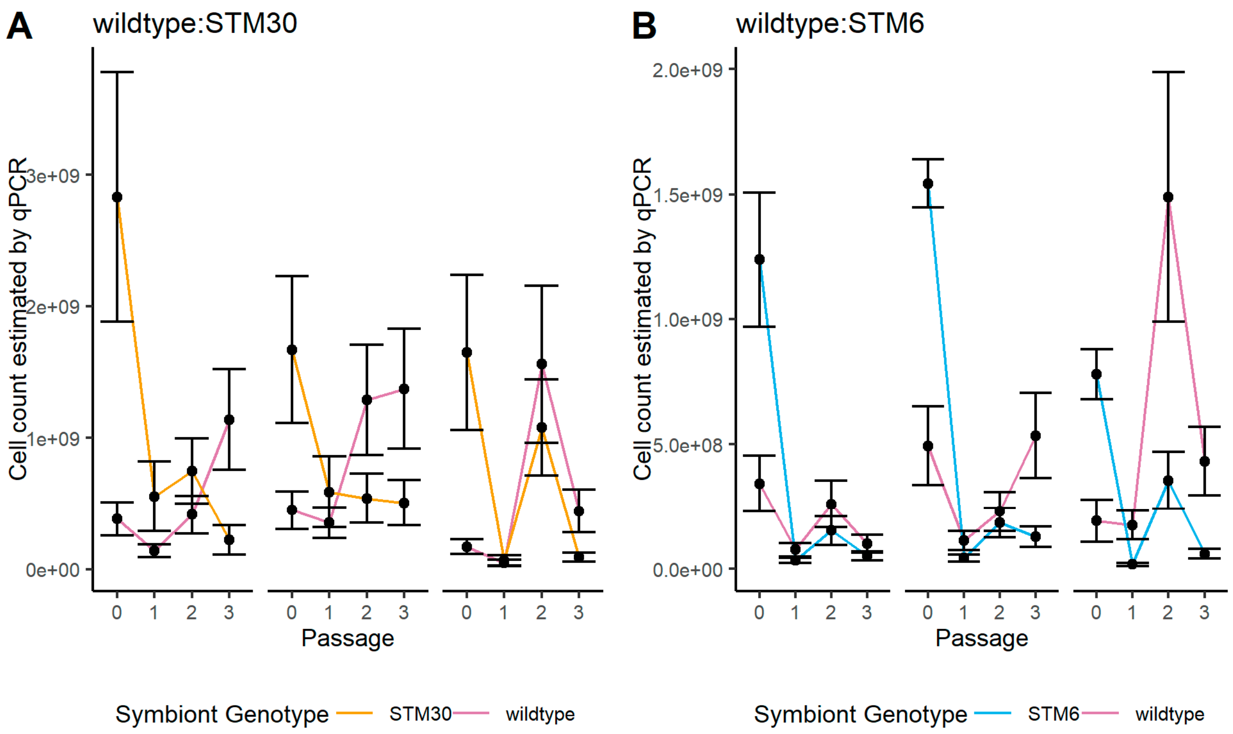

Figure S7. Estimated population sizes of wildtype co-inoculated with either mutant over serial exposure to *in planta* growth in Magenta Boxes. The wildtype was co-inoculated in three independent lineages with STM30 (A) or STM6 (B), both of which are less beneficial than the wildtype. Seedlings were inoculated with a high concentration of rhizobia that favored the mutant genotype. The population size of each genotype was estimate using qPCR at four different time points: the initial inoculation and after passage 1, 2, and 3 of *in planta* growth. Error bars indicate standard error of qPCR technical replicate estimates.

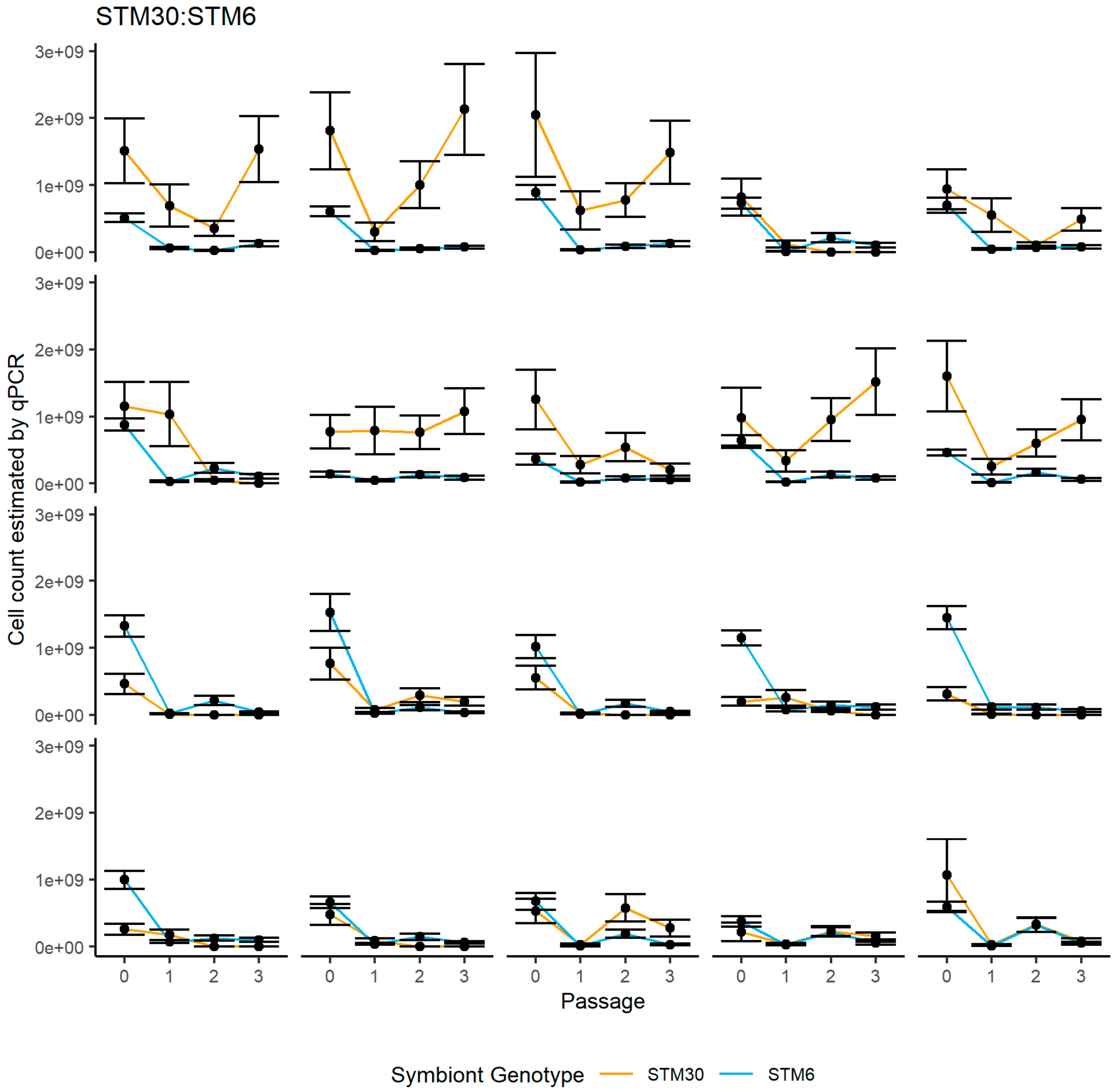

Figure S8. Estimated population sizes of both mutants co-inoculated together over serial exposure to *in planta* growth in Magenta Boxes. STM30 and STM6 were co-inoculated in 20 independent lineages. Seedlings were inoculated with a high concentration of rhizobia that ranged in initial relative abundance. The population size of each genotype was estimate using qPCR at four different time points: the initial inoculation and after passage 1, 2, and 3 of *in planta* growth. STM30 provides more benefit than STM6 in clonal inoculation. Error bars indicate standard error of qPCR technical replicate estimates.

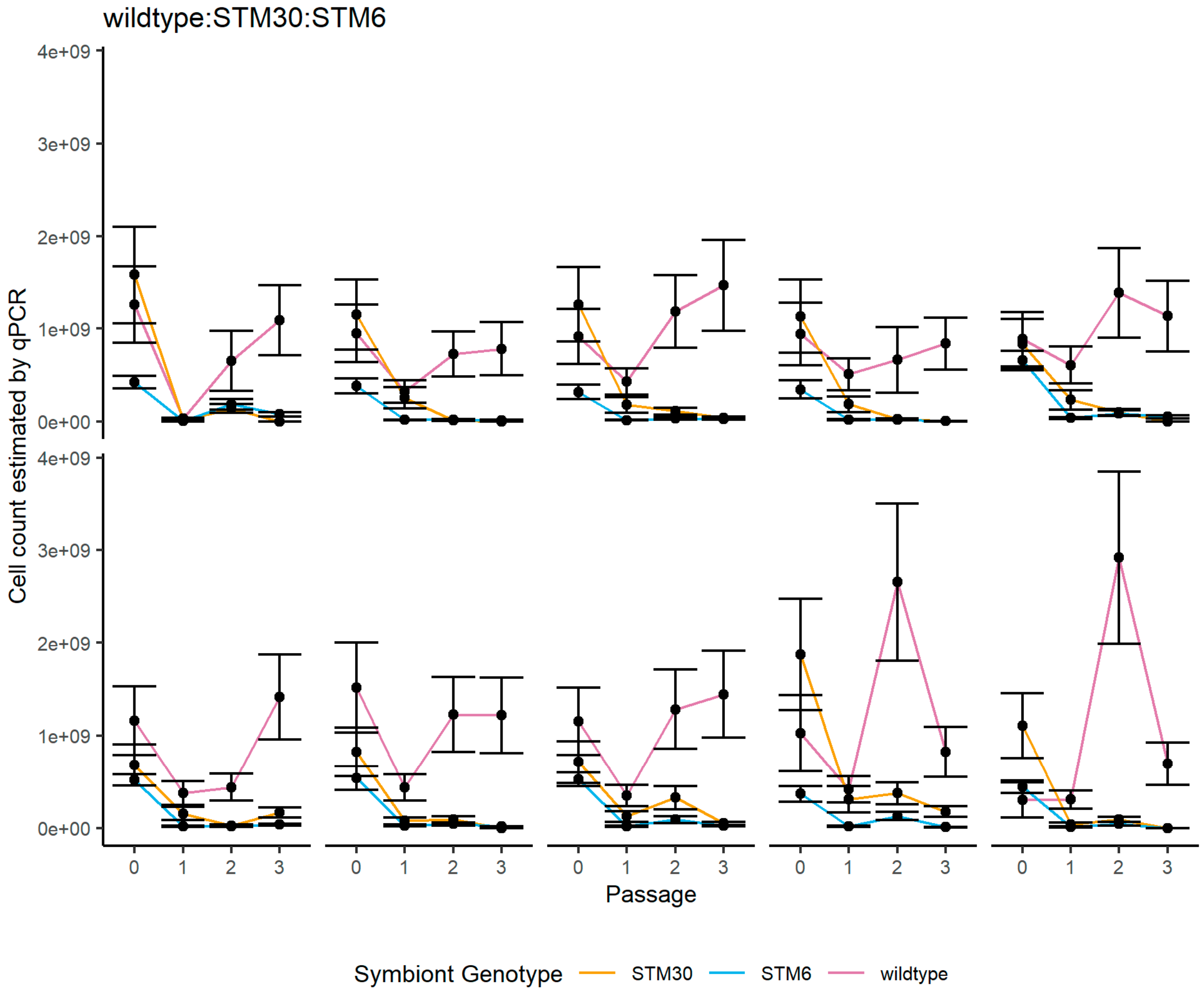

Figure S9. Estimated population sizes of all three genotypes co-inoculated together over serial exposure to *in planta* growth in Magenta Boxes. Wildtype, STM30, and STM6 were co-inoculated in 10 independent lineages. Seedlings were inoculated with a high concentration of rhizobia that ranged in initial relative abundance. The population size of each genotype was estimate using qPCR at four different time points: the initial inoculation and after passage 1, 2, and 3 of *in planta* growth. The order of benefit provided by rhizobial symbionts in wildtype > STM30 > STM6. Error bars indicate standard error of qPCR technical replicate estimates.
